# Special Issue “2nd Edition: Advances in Molecular Simulation”

**DOI:** 10.3390/ijms24021491

**Published:** 2023-01-12

**Authors:** Małgorzata Borówko

**Affiliations:** Department of Theoretical Chemistry, Institute of Chemical Sciences, Faculty of Chemistry, Maria Curie-Skłodowska University in Lublin, 20-031 Lublin, Poland; borowko@hektor.umcs.lublin.pl

Molecular simulation is becoming a standard tool for researchers working in different fields, such as physics, chemistry, material science, biology, medicine, engineering, and many others. Simulations provide information that cannot be obtained from experiments and offer scientists new cognitive opportunities. Advanced computational methods are used for a rational design of new chemical compounds, macromolecules, nanoparticles, and more complex assembled structures at any level of organization, from molecular to viruses.

A major goal of this Special Issue is to show a wide range of applications of molecular simulations and to draw together contributors from different disciplines to stimulate the exchange of knowledge and experiences. The collection of articles gathered here can be divided into two groups. The first group concerns the modeling of the internal structure of macromolecules and interactions between different complex chemical structures. The second represents typical physico-chemical studies systems containing many molecules.

Most works belong to the first group, which to some extent reflects the general trend in molecular sciences. The research is focused on specific chemical compounds with potential applications in medicine. In particular, the studies were aimed at improving the treatment of serious diseases, especially cancer, and preventing antibiotic resistance. Other articles are devoted to pesticide resistance and biomembrane modeling.

Tran et al. describe the effects of alternative one-point mutations on the New Delhi Metallo β-Lactamase-1 resistance carbapenem antibiotics and β-Lactamase inhibitors. Antibiotic resistance is becoming more and more critical due to bacteria’s evolving hydrolysis enzymes. Variant strains can strongly affect the resistance of bacteria producing NDM-1. The goal of the study is to identify which mutants could change the effectiveness of antibiotics and β-lactamase inhibitors toward bacteria. Using molecular docking, free energy calculations, and molecular dynamics simulation they analyze a wide range of complexes based on the PAM-1 matrix. Amelia and co-workers focus on the computational prediction of resistance-induced alanine-mutation in ATP site of epidermal growth factor receptor. The epidermal growth factor receptor resistance to tyrosine kinase inhibitors can cause low survival rates in mutation-positive non-small cell lung cancer patients. It is necessary to predict new mutations in the development of more potent inhibitors to enhance the effectiveness of the therapy. Four mutations that could be anticipated in the development of the next inhibitor to overcome the incidence of resistance in lung cancer patients are theoretically identified. The work by Wojtkowiak et al. is part of mainstream cancer drug research. They employ quantum–chemical approaches to study chalcogen bond as a factor stabilizing ligand conformation in the binding pocket of Carbonic Anhydrase IX receptor mimic. The overexpression of this isozyme contributes to the acidification of the extracellular matrix which promotes the growth and metastasis of the tumor. Therefore, it is seen as an attractive drug target. The molecule acetazolamide is chosen as a ligand because of its anticancer properties. The developed models can be useful in the design of new inhibitors with desired pharmacological properties. In turn, Saeed and co-workers discuss the structure and properties of acetophenone-based 3,4-dihydropyrimidine-2(1H)-thione using experimental and theoretical methods. This compound is a potential inhibitor of tyrosinase and ribonucleotide reductase. The exploration of such inhibitors is an emerging field for the treatment of skin cancer to overcome chemoresistance and side effects of already available drugs. In comparison to the already reported tyrosinase inhibitors, the newly synthesized derivatives exhibited almost seven-fold better inhibition of tyrosinase. Using molecular dynamics simulations Rasngubpit et al. explore the binding of glutathione and s-hexyl glutathione to glutathione S-transferase from Rhipicephalus (Boophilus) microplus. This process is of great practical importance as these ticks cause huge occurrence losses in livestock production. The long-term use of acaracides enhances acaracide resistance. One of the mechanisms underlying acaracide resistance is an increase in the metabolic activity of glutathione S-transferase that catalyzes the conjugation of glutathione to insecticides causing an easy-to-excrete conjugate. The study provides a deep insight into the insecticide-resistance mechanism. Bizzarri analyzes the conformational heterogeneity of selected variants of the tumor suppressor p53 using the modeling and all-atom molecular dynamics simulations. Four different punctual mutations, which are known to affect DNA binding and belong to the most frequent hot-spot mutations in human cancers, are considered. The article by Rasheed and coworkers concerns the search for potent uerase inhibitors. Urease is an amidohydrolase enzyme that is responsible for numerous diseases in humans. The Authors report the synthesis of a set of 1-aroyl-3-[3-chloro-2-methylphenyl] thiourea hybrids with aliphatic and aromatic side chains as well as the careful analysis of the products. The study involves biochemical evaluation and various theoretical approaches, such as density functional theory, antioxidant radical scavenging assay, molecular docking, and molecular dynamics simulations. All identified urease enzyme inhibitors are more active than the standard ones. Xia et al. deal with the cloning and characterization of fructose-1,6-bisphosphate aldolase from Euphausia Superba. This enzyme is involved in glycolysis and gluconeogenesis. The structure of the cloned gene is discussed. Apart from standard experimental methods, bioinformatic analysis is performed. For molecular modeling, a hierarchical approach to structure prediction is used.

Biomembranes consist of lipids, proteins, and carbohydrates and are fundamental to life. They are responsible for the cell envelopes that enable cells to absorb nutrients and exclude the most harmful agents from entering cells. The protein-lipid interactions are crucial for these processes. The work by Chen et al. concerns the archaeal lipids regulating the trimeric structure dynamics of bacteriorhodopsin for efficient proton release and uptake. They carried out long-term all-atom molecular dynamics simulation with the single-point mutagenesis to delineate the molecular mechanism of the specific interaction of the considered archeal lipids with bacteriorhodopsin during the proton release and uptake processes and found that these lipids are essential for stabilizing the bacteriorhodopsin trimer and maintaining the coherent conformational dynamics necessary for proton transfer.

The second group of works represents typical physico-chemical studies of systems consisting of a great number of particles. These articles concern simulations of the following phenomena: (i) adsorption on special substrates, (ii) phase transitions, and (iii) self-assembly.

Raza and co-workers performed molecular dynamics simulations to study the adsorption behavior of hydrogen, carbon dioxide, and methane on four sub-models of kerogens of varying thermal maturities over a wide range of pressures and temperatures. These simulations have a purely practical aspect namely they show that the organic-rich shales can be used for competitive $H_{2}$ and $CO_{2}$ storage. Thus, the study contributes to carbon/hydrogen economy strategies.

Phase transitions are the subject of two articles. Mareev and Potemkin present the molecular dynamics study of the morphology of silicon. They demonstrate an ultrafast reversible phase transition in silicon (from cubic diamond to β−Sn phase) under ultrafast pressure loading. Such a transition occurs only on the shock-wave front if pressure overcomes 11 GPa at the sub-ps timescale. Atomic volume, centrosymmetry, and the X-ray-diffraction spectrum are revealed as effective indicators of phase-transition dynamics. The latter constitutes a breakthrough in the path toward simple X-ray optical cross-correlation and pump-probe experiments. Nazarychev et al. systematically explore how the cooling rate affects the accurate description of the crystallization of paraffins in atomic-scale molecular dynamics simulations. They show that a certain threshold in the values of cooling rates exists. When cooling is slower than the threshold, the simulations qualitatively reproduce an experimentally observed abrupt change in the temperature dependence of the density, enthalpy, and thermal conductivity of paraffins upon crystallization. Beyond this threshold, when cooling is too fast, the paraffin’s properties in simulations start to deviate considerably from experimental data.

Self-assembly is the basis of various processes in nature and technology. Extremely complex structures can be built from small “bricks” via their assembly under precisely chosen conditions. Such a “molecular Lego” contains different blocks, from atoms, molecules, macromolecules, and nanoparticles to viruses and even greater objects. A series of three articles concerns the simulations of the assembly in different systems. Two articles are devoted to polymer-tethered (hairy) nanoparticles which are perceived as high-performance materials that combine the special features of cores and attached ligands. One work, however, refers to the formation of virus-like particles.

Borówko and Staszewski investigate the polymer-tethered nanoparticles in narrow slit-like pores using coarse-grained molecular dynamics simulations. The confinement in pores creates new possibilities for controlling the shape transformation of individual hairy particles and their self-organization. The impact of interactions with the surfaces and the wall separation on the system morphology is discussed. Different ordered structures, resembling two-dimensional crystalline lattices, are reported. The paper by Sato et al. provides fundamental insight into the sensing applications of polymer-tethered nanoparticles. They use the dissipative particle dynamics method to investigate the influence of selected parameters on sandwiching properties of Janus-type tethered nanoparticles. Nanosensors are beneficial devices that can convert microscopic information derived from the behavior of atoms and molecules into macroscopic data. For example, biomolecules can be sandwiched between nanoparticles and detected by surface-enhanced Raman scattering. Controlling the gap between extremely close nanoparticles and stably capturing the target molecule in the gap are crucial aspects of this strategy.

Macromolecular self-assembly is the basis of many phenomena in life and material sciences that find diverse applications in technology. A prominent example is the formation of virus-like particles that act as stable empty capsids used for drug delivery or vaccine fabrication. The virus-like particles are, in turn, protein assemblies. Depta et al. present the data-driven modeling approach for capturing macromolecular self-assembly on scales beyond traditional molecular dynamics while preserving the chemical specificity. Each macromolecule is represented by an anisotropic object and high-dimensional models are formulated to describe interactions between molecules and with the solvent. The self-assembly process is modeled as a combination of diffusive effects and pairwise interaction of molecules. The Kriging-based strategy building upon high-throughput molecular dynamics simulations with the Martini force-field is employed including semi-automated supervised learning to derive data-driven protein–protein interaction potentials. This generic method is applied to capture the formation of hepatitis B virus-like particles from the smallest building unit (the dimer of the core protein HBcAg). Assembly pathways and kinetics are analyzed and compared to the available experimental observations.

Taken together, all articles published reflect well the most interesting current directions in molecular simulation studies. It is the pleasure of the Guest Editor to thank all authors for their important contributions to this Special Issue.

